# Effect of Biochar Modification by Vitamin C, Hydrogen Peroxide or Silver Nanoparticles on Its Physicochemistry and Tetracycline Removal

**DOI:** 10.3390/ma15155379

**Published:** 2022-08-04

**Authors:** Agnieszka Tomczyk, Katarzyna Szewczuk-Karpisz

**Affiliations:** Institute of Agrophysics, Polish Academy of Sciences, Doświadczalna 4, 20-290 Lublin, Poland

**Keywords:** detoxification, antibiotics removal, chemical treatment, agricultural biochar, sorption efficiency

## Abstract

Chemical modification of biochars can improve their adsorption capacity relative to antibiotics, posing a serious threat to the environment. Therefore, this research is aimed at the treatment of sunflower husk biochar (BC) by vitamin C, hydrogen peroxide or silver nanoparticles and the impact of this procedure on the biochar porosity, surface chemistry, and ability to remove tetracycline (TC). During the study, BC was produced by pyrolysis of sunflower husks at 650 °C. All solids were characterized using potentiometric titration, nitrogen adsorption/desorption, Fourier transform infrared spectroscopy, etc. The experimental adsorption data was described by kinetics equations: pseudo-first order, pseudo-second order, and particle internal diffusion (IPD) models as well as by isotherms of Langmuir, Langmuir-Freundlich, and Redlich-Peterson. The obtained results indicated that the biochar upgraded by vitamin C (BCV) had the highest ability to attract antibiotic molecules and, as a result, the TC adsorption on its surface was the largest. Furthermore, the TC desorption from this material was minimal. The measured TC adsorbed amounts for the modified BCs were as follows: 47.75% (7.47 mg/g) for BCV, 37.35% (8.41 mg/g)-for biochar treated by hydrogen peroxide (BCH), and 42.04% (9.55 mg/g) for biochar modified by silver nanoparticles (BCA). The lowest adsorption level was noted for non-modified biochar, i.e., 34.17% (6.83 mg/g). Based on the presented results it can be stated that the upgraded biochars had a good potential to improve the tetracycline removal from aqueous media, e.g., groundwater.

## 1. Introduction

Currently, approximately 100–200,000 tones of antibiotics are consumed annually worldwide [1]. These compounds are used, inter alia, in agriculture. They are ingredients of feed and veterinary drugs [2], the use of which allows to keep animals in crowded areas. Numerous drugs (including tetracycline (TC), sulfonamides) are not metabolized 100% by organisms [1]. As a consequence, their large amounts end up in various ecosystems through feces, where they pollute water and soil as well as contribute to the formation of dangerous, drug-resistant strains of microorganisms [3]. Tetracycline is one of the most widely used antibiotics. It is produced by *Streptomyces* microorganisms, and thus included in the group of drugs of natural origin (together with chlortetracycline and oxytetracycline). Due to high efficiency of TC in organism curation, its application is massive. Besides the ability to inhibit the growth of pathogens, it can also promote feed utilization and improve animal growth rate [4]. Because of the fact that even 70–90% of TC applied to animals are excreted in urine or feces in an antimicrobial active form [5], this substance was considered as posing a serious risk to the environment.

There are many well-known methods of TC removal. There are heterogeneous photocatalysis, ozonation, fenton, and many others. Heterogenous photocatalysis can be performed using Bi_24_O_31_Br_10_ nanosheets, which enables the degradation of 95% of TC under UV light irradiation. Ozonation for 4–6 min allows up to100% of TC to degrade. In turn, tetracycline removal by fenton can be about 24 mg/L [6]. Unfortunately, all these methods have many disadvantages, and they can generate free radicals and cause secondary pollution of the environment [6]. This led many researchers to focus on the adsorption phenomenon. So far it has been reported on several occasions that adsorption is a simple, low-cost, easy to operate, eco-friendly, and efficient technique for the removal of numerous pollutants, including antibiotics. The group of adsorbents widely used for TC removal includes activated carbon, carbon nanotubes, graphene oxide, nanocomposites, polymeric resin, and biochar. The application of these substances can affect the rate of TC in the soil and water, i.e., its bioavailability and mobility [6].

Recently, biochar (BC) was one of the most popular adsorbents used to remove pollutants. It is defined as a fine grained, carbon-rich material, obtained from biomass by pyrolysis. The group of substrates applied for biochar production is highly diverse, and includes energy crops, forest waste, agricultural (plant and animal) waste, sewage sludge, the organic fraction of municipal waste or residues from agri-food processing. The choice of biomass depends on the purpose of the produced biochar [7]. Biowaste from the food industry and agriculture is the largest source of feedstock for pyrolysis [8]. According to the report of International Solid Waste Association [9], global agricultural waste generation stands at 7–10 billion tons per year, and only 30–40% of them are collected properly. The remainder are just thrown away. Sunflower husks are an example of biowaste generated mainly in the food industry. In some cases, they are used as a feed for farm animals as well as valuable raw material for many industrial products [10]. But most of them are usually discarded. In the 2018–2019 season, world sunflower seed resources were equal to 56.1 million tons [11]. They were higher by 6% than in the previous season. In 2015–2016, total domestic use of sunflower seeds in Europe amounted to 8.1 million tons, whilst in 2014–2015 it was 8.9 million tons [11]. All this means that sunflower husks are generated in large amounts, and they require proper management. It is necessary to find the most effective way to utilize the excess of these materials. The production of biochar and its subsequent use as an adsorbent can be a perspective solution of the above problem. This is a low-cost and eco-friendly conception of biomass management. Due to specific properties, such as the high content of organic carbon and mineral substances in stable forms, developed porosity, and specific surface area, biochar can be successfully used in many branches of the economy. The application of BC in agriculture has been known for centuries and performed in Asian countries, including Japan and China [12]. The BC use in removing various types of pollutants during wastewater treatment or remediation of degraded soils is also extremely important [7,13].

In order to improve biochar adsorption capacity relative to organic and inorganic compounds, many scientists performed studies on its modifications [14]. They carried out BC activation using physical or chemical methods [6]. Usually, modified biochars have higher potential in removing hazardous substances than ‘raw’ materials [15,16]. Several scientists have carried out biochar modifications and measured the adsorption capacity of the resulting materials against tetracycline. For example, the research conducted by Zhou et al. [17] showed that modification of biochar by ZnCl_2_ and FeCl_3_·6H_2_O improved its adsorption capacity towards TC from 20 mg/g to 75 mg/g at pH 5.0. The study conducted by Dai et al. [15] indicated that magnetic modification of biochar led to its enhanced adsorption capacity relative to TC to be equal to 98.33 mg/g. Shen et al. [18] demonstrated that manganese dioxide caused an increase in maximum adsorption capacity of Chinese herbal medicine residues-biochar towards TC to the value of 131.49 mg/g. Additionally, Tan et al. [19] reported that H_2_O_2_ modified biochar exhibited higher adsorption capacity in relation to the selected antibiotic (43.45 mg/g) than non-modified biochar (32.0 mg/g).

The authors of this paper have also performed the production of biochar from biowaste, and they conducted modifications of obtained carbon-rich material to improve its adsorptive efficiency in tetracycline removal. In this way, a comprehensive research on the effects of modification, both inorganic and organic, on biochar adsorptive features relative to hazardous drug was performed. The investigation aimed at four main objectives: (i) determination of the effects of inorganic/organic modification on the physicochemical properties of biochar; (ii) investigation on the kinetics and equilibrium isotherms of TC adsorption onto biochar; (iii) calculation of the biochars efficiency in TC adsorption in aqueous media; and (iv) evaluation of the influence of pH and desorbing agent type on TC desorption from biochars. The authors predict that modification of biochars will improve TC removal, and the modified materials will prove useful in practical applications, especially in the removal of antibiotics from groundwater.

## 2. Materials and Methods

### 2.1. Production of Biochar and Chemically Upgraded Biochars

Biochar (BC) was produced by the Czestochowa University of Technology via a 15-min process of autothermic pyrolysis of sunflower husks at 650 °C, under oxygen-limited conditions. This process was performed in the pilot lab-scale reactor working under pressure and flow conditions that ensured the maximum heating rate of the fragmented biomass. More details of the biochar preparation are given in Gluba et al. [20]. The pyrolysis products were air-dried, crushed and sieved through a 2 mm mesh. Moreover, they were washed three times by distilled water to remove impurities.

The chemically upgraded biochars silver nanoparticles-biochar (BCA), vitamin C-biochar (BCV), and hydrogen peroxide-biochar (BCH), were prepared in two steps. The proportion of BC powder to reagents was 1:30 (*v*/*v*). The reagents: L-ascorbic acid (CAS Number: 50-81-7), hydrogen peroxide (CAS Number: 7722-84-1), nitrate silver (CAS Number: 7761-88-8), and PVP (CAS Number: 9003-39-8) were purchased from Sigma-Aldrich (Burlington, MA, USA). They were used for biochar modifications at the following concentrations: 500 mg/L–silver nanoparticles, 1 mol/L–vitamin C and 30%–H_2_O_2_. The silver nanoparticles (Ag-NPs) were obtained by the chemical reduction method [21]. Silver nitrate (AgNO_3_) was used as a silver nanoparticle precursor, ascorbic acid–as a reducing agent, and polyvinyl pyrrolidone (PVP)–as a stabilizer. First, the metal precursor was added to the PVP aqueous solution, and the mixture was heated to 80 °C. Next, the second solution was prepared from PVP and ascorbic acid. The chemical reduction took place as a result of the dropwise addition of the solution with reducing agent to the solution with metal precursor. The solution was stirred for 1 h at constant temperature (80 °C), then it was cooled and kept in a refrigerator at 5 °C. The Ag-NPs had a diameter of 46 nm. The size of Ag-NPs was analyzed by a CPS analyzer (CPS Instruments, Anaheim, CA, USA).

At the first step of biochar modification, sunflower husk biochar was mixed with the selected solutions and treated with ultrasound waves for 15 min (VGT-1860QT, GT Sonic, Guangdong, China). Next, the suspensions were stirred for 24 h at room temperature (20 ± 1 °C) (30 rpm, Multi RS-60, Biosan, Riga, Latvia). At the end of this, the modified biochars were collected by filtration of the suspensions, washed three times with deionized water, and air dried.

### 2.2. Physicochemical and Surface Properties of Biochars

The moisture content (M) of biochars was determined by weighing the sample into an aluminium weighing tin and drying in an oven overnight at 105 °C. This parameter was calculated based on the loss of weight of the sample using:

(1) $$M\;\left[\%\right]=100\cdot \frac{\mathrm{WAR}-\mathrm{WOD}}{\mathrm{WOD}}.$$

where WAR is the weight of the sample [g], and WOD is the weight of the oven-dried sample [g].

The ash content was calculated by weighing the residue after 6 h of combustion at 750 °C in a muffle furnace (Czylok FCF12SP, Jastrzębie-Zdrój, Poland). The total organic carbon (TOC) content [%] was measured with a TOC-VCPH analyser (Shimadzu, Kyoto, Japan).

The content of surface acidic and basic functional groups was determined by the Boehm method [22]. This method is based on the assumption that the constants of acidic and basic groups differ by several orders of magnitude and, as a result, it is possible to neutralize them using appropriately selected compounds and distinguish them by acid-base titration. At the beginning, the suspensions were prepared using 0.1 g of biochars and 10 mL of the following reagents: 0.1 mol/L HCl and 0.1 mol/L NaOH. The mixture was stirred for 24 h. After filtration, phenolphthalein was added as an indicator to 5 mL of the filtered solutions. Next, the solutions were titrated with 0.1 mol/L NaOH solution or 0.1 mol/L HCl solution. The following formula was used to calculate the content of groups:

(2) $$A_{x}=\frac{\left(V_{\mathrm{tit}}-V_{x}\right)\cdot C_{\mathrm{tit}}\cdot X}{m_{x}}$$

where V_tit_–the volume of the titrant used to titrate the control sample [mL], V_x_–the volume of the titrant used to titrate the sample [mL], C_tit_–the titrant concentration [mol/L], X–the ratio of initial volume of suspensions/used volume of suspensions, m–the sample weight [g].

The surface charge density (*σ*_0_) of modified and non-modified BC was obtained based on the potentiometric titration data. The titration was performed in the pH range of 3–10, using an automatic burette (Titrino 702 SM, Metrohm, Herisau, Switzerland) and 0.1 mol/L sodium hydroxide (NaOH) as the titrant. The probes were prepared by the addition of 0.1 g of BC to 20 mL of supporting electrolyte–0.001 mol/L CaCl_2_. The *σ*_0_ parameter was calculated using the following equation [23,24]:

(3) $$\sigma_{0}=\frac{\Delta V\cdot C\cdot F}{m\cdot S_{\mathrm{BET}}}$$

where ΔV–the difference in the base volume added to the suspension and the supporting electrolyte, leading to the selected pH value (mL); c–the NaOH concentration (mol/L); F–the Faraday constant; m–the weight of BC in the suspension (g); and S_BET_–the specific surface area of BC (m^2^/g).

Nitrogen adsorption/desorption isotherms obtained at 77 K was used to determine the biochar specific surface area (S_BET_), total pore volume (V_t_), total micropore volume (V_m_), and average pore diameter (D), using the Brunauer-Emmett-Teller (BET) and Barrett, Joyner and Halenda (BJH) methods. The analyses were performed using the ASAP 2420 apparatus (Micromeritics, Norcross, GA, USA). Before the analyses, all biochars were submitted to degasification at 105 °C for 12 h.

The FTIR spectra of biochars were determined by FTIR Spectrometer (Nicolet 8700A FTIR spectrometer, Thermo Scientific, Somerset, NJ, USA). Biochars were prepared in the form of pellets composed of a homogeneous mixture of 0.5 mg finely grounded biochar and 100 mg powdered KBr. The FTIR spectra of biochars were recorded in the range of 400–4000 cm^−1^. The H/C and O/C ratios and (O+N)/C were analysed with a Leco TruSpec CHNS (St. Joseph, MI, USA) elemental analyzer. Based on the chemical compositions of biochars, the higher heating value (HHV) and energy density (ED) [25] were calculated using:

(4) $$\mathrm{HHV}\;\left[\frac{\mathrm{MJ}}{\mathrm{kg}}\right]=(\left(0.338\cdot C\%\right)+1.428\cdot \left(H\%-\frac{O\%}{8}\right)+\left(0.095\cdot S\%\right))$$

(5) $$\mathrm{ED}\;\left[\%\right]=\frac{\mathrm{HHV}\;\mathrm{BC}}{\mathrm{HHV}\;\mathrm{Sunflower}\;\mathrm{husks}}$$

where C%–the carbon content; H%–the hydrogen content; O%–the oxygen content; S%–the sulphur content.

### 2.3. Batch Adsorption/Desorption Experiment

The samples, prepared by the addition of biochar to the solution containing TC and the supporting electrolyte, were mixed for the specific time: 5–1440 min for kinetics and 24 h for equilibrium adsorption, with a rotator (30 rpm, Multi RS-60, Biosan, Riga, Latvia), under constant pH conditions, at room temperature (21 °C ± 2 °C). The weight of biochar used in the sample preparation was established based on the previously determined dependency of biochar weight on tetracycline removal efficiency. In turn, the time necessary to reach equilibrium in the systems was determined based on the study on TC adsorption as a function of time. The concentrations of TC were: 75 mg/L for kinetics study and 1–100 mg/L for equilibrium measurements. The pH value of system was 5.0 ± 0.5, and the ionic strength of the background solution was maintained using supporting electrolyte—0.001 mol/L CaCl_2_. After the adsorption completion, the suspensions were filtered by paper (390, Ahlstrom Munktell, Bärenstein, Germany) and syringe filters (nylon, 0.45 µm, Thermo Fisher Scientific, Waltham, MA, USA). The TC concentration in the solutions was determined using high-pressure liquid chromatography (Ultimate 3000, Dionex, Thermo Fisher Scientific, Waltham, MA, USA) equipped with DAD (diode-array) detector and column Hypersil Green PAH (Thermo Fisher Scientific, Waltham, MA, USA). The elution was gradient. Acetonitrile:water mixture in the ratio of 4.5:5.5 was used during the first 5 min. After that, the concentration of acetonitrile was increased to the value of 100%. From 9 to 11 min, the eluent was 100% acetonitrile, and later its concentration was reduced to 45%. A single analysis lasted 15 min, and the injection volume was 10 µL. The column temperature was 30 °C. The flow rate was 1 mL/min. The HPLC apparatus cooperated with the software ‘Chromeleon’. The limit of the TC detection was 1 μg/L. The TC adsorbed amount (mg/g) was calculated as follows:

(6) $$q_{e}=\frac{\left(C_{0}-\;\mathrm{Ce}\right)\cdot V}{m},$$

where C_0_ is the initial concentration (mg/L), C_e_ is the TC concentration at equilibrium (mg/L), m is the mass of the sample (mg), and V is the volume of the solution (L).

The process of desorption was conducted with distilled water of different pH value, i.e., 5, 7 and 9, 0.1 mol/L HCl, and 0.1 mol/L NaOH. At first, the adsorption process was conducted in the identical way as described in the previous part. The concentration of TC was 100 mg/L and the time was 24 h. After the process completion, the suspensions were centrifuged (centrifuge MPW-223e, Warsaw, Poland, 10 min, 33,540× *g*) and, after the supernatant removal, the biochars were inundated with 10 mL of water. The desorption process was conducted for 1 h in a rotator (30 rpm, Multi RS-60, Biosan, Riga, Latvia). The amount of desorbed antibiotic was calculated based on the difference between the amount of TC determined in the solution after the desorption experiment and the amount of TC adsorbed on the biochars.

### 2.4. Modeling of Adsorption Data

The obtained adsorption data was described by kinetics equations: the pseudo-first order (Equation (7)), the pseudo-second order (Equation (8), and particle internal diffusion (IPD) (Equation (9)) models, and by isotherms: Langmuir (Equation (10)), Langmuir-Freundlich (Equation (11)), and Redlich-Peterson (Equation (12)). The calculations were made using SciDavis software. The all used equations are shown in Table 1.

### 2.5. Statistical Analysis

All measurements were made in triplicate, and the points of graphs were obtained from the averaged data. The measurement errors were calculated using the Statistica software (13.0, TIBCO Software, Palo Alto, CA, USA), SciDavis software (2.7, Tilman Benkert and Knut Franke, Boston, MA, USA), and Microsoft Office software (MS Office 2010, Microsoft, Redmond, WC, USA). The Tukey test for obtained results was *p* < 0.05.

## 3. Results and Discussion

### 3.1. Characteristic of Biochar

Table 2 presents physicochemical parameters determined for sunflower husks (biomass) and biochar produced from it. Additional results of the surface and physicochemical analyses are summarized in the publication of Gluba et al. [20].

The obtained results showed that after pyrolysis there was a decrease in moisture (9.38% to 0.49%) as well as an increase in ash content (2.43 to 35.4%) of the material. This was a result of biomass dehydration and combustion [7]. The HHV and ED are important parameters indicating the energetic potential of biomass from different sources [32]. In the case of the examined biomass and biochar, the HHV parameter increased more than twice after the pyrolysis, and the calculated energy density was above 2%. This means that the biochar from sunflower husks has high heating value and energy density, and it can be considered as a product of good energetic potential [32]. Saleh et al. [33] documented that the HHV of biochar obtained from sunflower husks at 450 °C was 14.91 MJ/kg, and ED was 1.11%. Yue et al. [34] presented that HHV of biochar produced from sunflower straw at 700 °C was 23.3 MJ/kg, and ED was 1.39%.

The study on elemental composition indicated that the ratios: H/C, O/C, and (N+O)/C decreased after pyrolysis. This is equivalent to the reduction in polarity and increase in aromaticity of the material, which usually improves the adsorption capacity of carbonaceous adsorbents [7]. Furthermore, the elemental ratio of H/C is used to evaluate the degree of carbonization and aromaticity of the biochar, and it is linked to the long-term stability in the environment [35].

**Figure 1:**
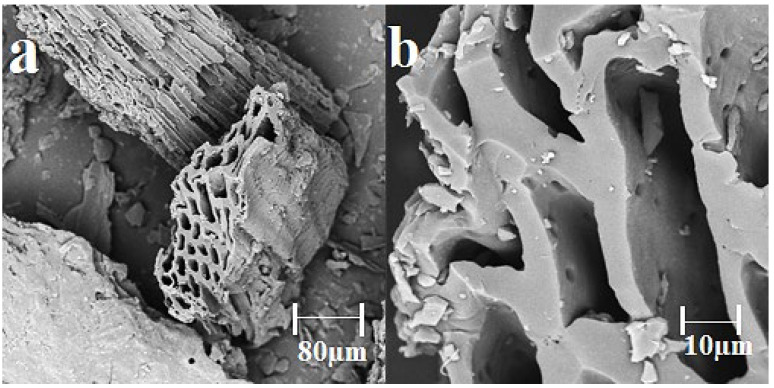
The SEM images (**a**) ×1000 and (**b**) ×8000 of biochar derived from sunflower husks.

The morphology of the obtained biochar was examined using a scanning electron microscope. The obtained images are presented in Figure 1.

The SEM analyses showed that the sunflower husk biochar had a heterogeneous structure rich in cracks, crevices, and channels. It is typically for biochar obtained by pyrolysis at high temperatures [7]. Other researchers found similar results [36,37,38,39]. For example, Shaaban et al. [36] explored the effect of pyrolysis temperature on biochar surface, and they reported that the high temperature of pyrolysis contributed to the formation of cracks and holes. The surface morphology of the tested sunflower husk biochar is comparable to those described by Suárez-Hernández et al. [38] and Jinsha et al. [39].

### 3.2. Impact of Organic/Inorganic Modifiers on Biochar Properties

#### 3.2.1. BET and Surface Chemistry Analysis

Table 3 presents the textural parameters determined based on nitrogen adsorption/desorption analyses as well as the results of TOC measurements and Boehm’s titrations of modified and non-modified biochars.

The results of BET analyses showed that both organic and inorganic modifications of the biochar reduced its specific surface area, micropore volume, and total pore volume. It is caused by the presence of ascorbic acid molecules and silver nanoparticles on the biochar surface as well as its oxidation by H_2_O_2_. The above substances block some pores of BC and make them inaccessible for N_2_ adsorption. The average pore radius of biochars has also changed after the modification. There was a great increase to the values of 36.37 nm and 17.68 nm for BCV and BCA, respectively. In the case of BCH, this change was not so large and D parameter was equal to 5.11 nm. These results indicated that the performed modification significantly influenced the textural parameters of biochar.

The chemical modification caused slight changes in TOC. A small increase in TOC was noted for BCV (from 82.15% to 82.92%) and for BCA (from 82.15% to 82.64%). In turn, for BCH a slight decrease was observed (from 82.15% to 80.72%). These results proved that silver nanoparticles and ascorbic acid enriched biochar with additional organic substances.

The content of acidic and basic groups in BC was also affected by the conducted modifications. These procedures increased the amount of acidic groups to the values of 3.10 mmol/g for BCV, 5.20 mmol/g for BCH, and 4.00 mmol/g for BCA, as well as the content of basic groups to the value of 3.40 mmol/g for BCV, 6.9 mmol/g for BCH, and 5.20 mmol/g for BCA. This indicated that all performed modifications changed the surface chemistry of biochar. A similar phenomenon was also observed by Huff and Lee [40]. The greatest changes noted for H_2_O_2_-biochar were dictated by the solid surface oxidation. In turn, the increase in the amount of acidic and basic groups on the biochar surface after the AgNPs modification was caused by the presence of PVP in the Ag nanoparticles. The Ag-NPs were adsorbed on the surface of biochar by PVP, which had pyridine groups (basic groups) and carbonyl groups (acid groups) in its structure.

#### 3.2.2. FTIR Analysis

**Figure 2:**
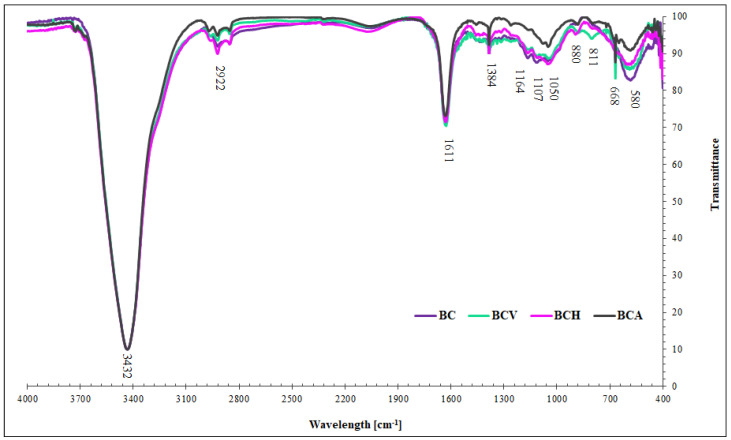
The FTIR spectra of biochars.

The results of FTIR analyses of modified and non-modified biochars are presented in Figure 2.

The FTIR spectra indicated that the surface of the tested biochars was dominated by functional groups of oxygenated hydrocarbons, i.e., carbohydrate structures of cellulose, hemicelluloses, and lignin [7]. All bands are summarized in Table 4. The differences between the spectra of modified and non-modified biochars were subtle.

For all biochars, the intensity of the band attributed to -OH groups was similar. The band corresponding with C-H stretching was the highest for BCH, whilst the band attributed to the stretching of aromatic C=C and C=O in carboxylic and lactonic groups was the highest for BCV. For BCV and BCH, the intensity of the band attributed to N-O stretching of pyridinic-N oxide was the highest. In turn, BCA showed the lowest intensity of the band, which was assigned to C-N stretching of secondary amines and C-H stretching of aromatic structures. The band attributed to aromatic C-H stretching was the highest for BCA and BCV, while the band corresponding with chloride and the CO_3_=groups was the lowest for non-modified BC. All these results indicated that the type of modifier applied for the BC modification strongly determined the chemistry of the BC surface. Chemical modifiers caused changes in the surface chemistry: ascorbic acid increased the aromaticity of the biochar surface, while BCH and Ag-NPs increased the nitrogen content and aromaticity of the biochar surface.

#### 3.2.3. Surface Charge Density Analysis

**Figure 3:**
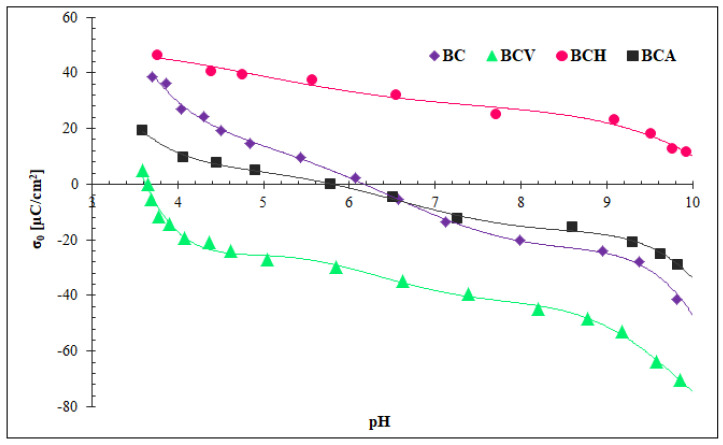
Influence of biochar modification on its surface charge density (σ_0_).

Figure 3 showed the results of potentiometric titration, i.e., the surface charge density of modified and non-modified biochars as a function of pH value.

The results showed that the organic and inorganic modification clearly influenced the surface charge density as well as the points of zero charge (pH_pzc_) of biochars. The pH_pzc_ parameter of non-modified biochar was 5.8, which meant that at pH 5, in which the adsorption study was performed, its surface was positively charged. The biochar modification with ascorbic acid resulted in a decrease in surface charge; the BCV surface was negatively charged in almost all examined pH ranges (3.7–10). During the modification, vitamin C was adsorbed on the BC surface based on hydrophobic interactions. Its molecules contain aromatic rings, which are arranged in π-π interactions with the aromatic rings of biochar. At pH > 4.10, ascorbic acid is deprotonated (pK_a_ = 4.1), and its negatively charged hydroxyl groups are probably responsible for the negative surface charge of carbonaceous material [43]. Modification with Ag-NPs resulted in minimal changes in surface charge density of BC, which were probably connected with the interaction PVP (a nanoparticle stabilizer) with the BC surface. The biochar modification with hydrogen peroxide caused a clear increase in biochar surface charge in the entire examined pH range. The pH_pzc_ point was even higher than 10. This was due to the oxidation of the biochar surface, resulting in the larger content of oxygen-containing groups as well as the protonation of the amino compounds of BC [19,44].

### 3.3. Adsorption/Desorption Experimental Study

#### 3.3.1. Dosage Effect

**Figure 4:**
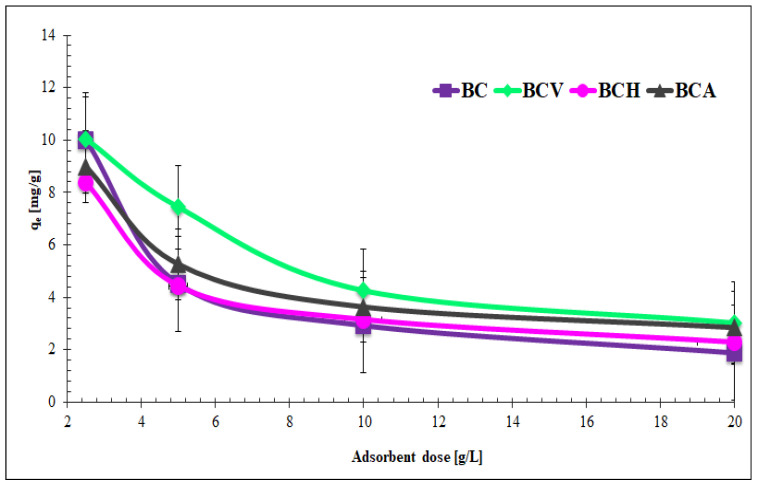
Effect of biochar dosage on tetracycline adsorption (C_0_ = 90 mg/L; time = 24 h; T = 20 °C; pH = 5).

Figure 4 presents the effect of the dose of modified and non-modified biochars on the tetracycline adsorbed amount.

The obtained results showed that the BC dose had a great influence on the antibiotic adsorbed amount. The adsorption of tetracycline decreased when the adsorbent dosage increased. This was mainly caused by the effect of flux splitting (i.e., concentration gradient) between the adsorbates and the biochars, which led to the reduction in the amount of TC adsorbed on the unit mass of biochar. Particle interactions, for example aggregation, may be another cause of the above phenomenon [40]. The particle aggregation leads to reduction in the adsorbent surface area, and it makes the length of the diffusion path larger [45]. Based on these experiments, the optimal dose of biochar for tetracycline adsorption was chosen to be 5 g/L.

#### 3.3.2. Contact Time Effect

**Figure 5:**
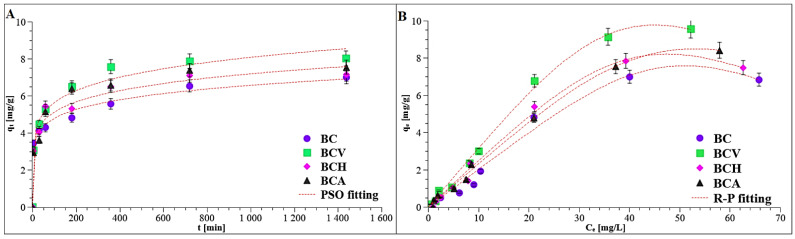
Adsorption kinetics (**A**) and isotherms (**B**) of TC on modified and non-modified biochars.

Figure 5A presents the time effect on the TC adsorption on modified and non-modified biochars as well as the fitting of experimental data to the selected theoretical models.

Initially, there was a rapid increase in the TC adsorption, while after 720 min, the plateau was reached (the thermodynamic equilibrium was established). This behaviour suggested strong interactions between active sites present on the biochars surface and the tracycline molecules. After 24 h, the adsorbed amount was 7.84 mg/g for BCV, 7.13 mg/g for BCH, 7.54 mg/g for BCA, and 6.99 mg/g for BC. Therefore, 24 h was chosen for the equilibrium adsorption measurements.

#### 3.3.3. Kinetics and Equilibrium Adsorption

Experimental kinetic data of TC adsorption on modified and non-modified biochars was analyzed using three models: PFO, PSO, and IPD. The obtained parameters are included in Table 5.

These results show that the PSO equation was better fitted to experimental results (R^2^>0.99) than the PFO one. The previous studies on tetracycline adsorption kinetics showed the same dependence [40,46]. The high R^2^ value obtained for the PSO equation suggested that chemisorption was mainly involved in TC bonding [27,46,47]. Based on the calculated rate constants, it was stated that the fastest adsorption occurred on BCV (*k*_2_ = 0.54 × 10^−2^ g/mg·min), whilst the slowest occurred on the non-modified biochar (*k*_2_ = 0.29 × 10^−2^ g/mg·min). The theoretical *q_e_* values obtained using PSO were very close to the measured experimental adsorption capacity (i.e., experimental *q_e_* for BCV was 7.84 mg/g, whereas theoretical one was 7.85 mg/g). The *b* parameter of IPD model indicates the thickness of the boundary layer. The higher its value, the greater the impact of this layer on the adsorption process. The performed data modeling using the IPD equation indicated that the *b* parameter had the greatest impact on the adsorption process on BCV, but the least on BC.

Figure 5B presents the plots of fitting of experimental data to the Redlich-Peterson isotherms. The parameters determined from the Langmuir, Langmuir-Freundlich, and Redlich-Peterson models are summarized in Table 5. The Redlich-Peterson isotherm yielded R^2^ > 0.98, which indicated that this isotherm should be used to describe experimental adsorption data. The Redlich-Peterson is a three-parameter isotherm connecting assumptions of the Langmuir and Freundlich isotherms. It assumes that the mechanism of adsorption is hybrid, and it does not follow ideal monolayer adsorption [30]. The Redlich-Peterson isotherm converges to the Henry law for β = 0 or to the Langmuir form when β = 1. For the examined systems, the exponent β was lower than 1, and thus multilayer adsorption of tetracycline was predominant. The calculated adsorption capacity was as follows: 6.83 mg/g for BCV, 7.47 mg/g for BCH, 8.41 mg/g for BCA, and 6.99 mg/g for BC. This means that the biochar modified with vitamin C was characterized by the highest adsorptive abilities relative to the selected antibiotic.

#### 3.3.4. Mechanisms of Adsorption

**Figure 6:**
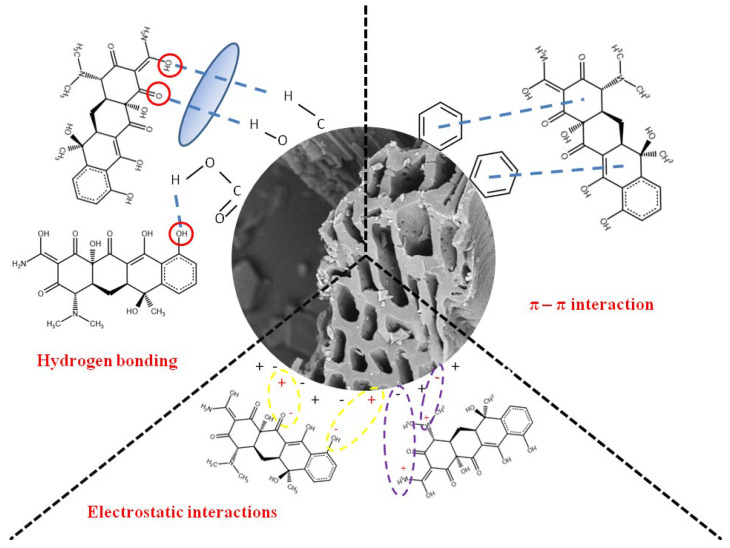
Mechanisms of TC adsorption on biochars.

The electrostatic interactions, hydrogen bonding, and π–π interactions between the adsorbent and the adsorbate might constitute the important roles in the adsorption mechanism of tetracycline on modified and non-modified biochars (Figure 6).

Tetracycline is a polar pollutant with amphoteric properties. It has three pK_a_ values (3.3, 7.7, and 9.7) and, as a result, a different charge at various pH values. The cationic form of TC occurs at pH < 3.3, zwitterionic–at 3.3 > pH > 7.7, and anionic–at pH > 7.7 [6]. The pH value of the systems, in which adsorption was conducted, was equal to 5. Under these conditions, the TC molecules were in the zwitterionic form. On the other hand, the surface charge of BC under selected pH conditions is strongly dependent on the modification type. At pH 5, non-modified biochar has surface charge density equal to +14 µC/cm^2^, BCV—−27 µC/cm^2^, BCA—+4.8 µC/cm^2^, and BCH—+40 µC/cm^2^. Generally, biochars can adsorb tetracycline via electrostatic attraction occurring between BC and TC fragments having charges of opposite signs. The π–π electron donor–acceptor interactions can also occur between graphite-like structures of biochar (donor) and aromatic rings of antibiotic (acceptor). TC has a strong electron-withdrawing ability by its ketone group and aromatic ring [40,48]. The last forces playing an important role in tetracycline adsorption are strong hydrogen bonds formed between functional groups of TC and biochars. The strength of the π–π interactions is strongly dependent on the surface chemistry of the adsorbent. The increased aromaticity of the material enhances this mechanism. The π–π electron donor–acceptor interactions are also important during the adsorption of dyes, e.g., methylene blue or red congo, on BC [49,50,51].

#### 3.3.5. Adsorption Efficiency of Biochars

The chemically upgraded biochars exhibited various efficiencies in TC removal from aqueous solution, which is dictated by the type of applied modifier. The calculated efficiencies were as follows: 47.75% for vitamin C-biochar, 37.35% for hydrogen peroxide-biochar, 42.04% for silver nanoparticles-biochar and 34.17% for non-modified biochar. Thus, it can be stated that all performed modifications of biochar enhanced its adsorptive ability. The inorganic and organic modification of biochars led to changes in their specific surface area and porosity, contents of surface functional groups and surface charge density. As was mentioned previously, the removal of tetracycline strictly depends on the BC properties listed above. Biochar modification with vitamin C contributed mainly to the increase in the content of aromatic structures, which are involved in the π-π electron donor-acceptor interactions between the adsorbent and the adsorbate. This change had the greatest effect in improving the sorption capacity of biochar in relation to tetracycline. Biochar modification with H_2_O_2_ was associated with an increase in the amount of acidic (lactonic, carboxylic and phenolic) and basic (N-containing) groups, which was reflected in Boehm’s titration results. Hydrogen peroxide can also make the basic groups protonated. In turn, the applied Ag-NPs were additional sites for the adsorption of tetracycline. The antibiotic molecules may interact with the nanoparticle stabilizer–PVP.

#### 3.3.6. Desorption

Table 6 shows the desorption degrees of TC from modified and non-modified biochars at pH 5, 7 and 9, as well as in the solutions of 0.1 mol/L HCl and 0.1 mol/L NaOH.

Desorption of tetracycline was strongly dependent on pH value. It was the largest under basic conditions. As was mentioned above, at pH 5 TC occurs in a zwitterionic form. As the pH increases, TC assumes a negative charge (i.e., anionic form). When the pH is getting higher, the surface of the biochar becomes more and more negative due to the dissociation of functional groups [34]. Then, electrostatic repulsion, occurring between the negative surface of biochar and anionic TC, significantly reduces the adsorbed amount of the antibiotic [6]. The migration of TC from the biochar surface toward the liquid phase is more favorable and, as a consequence, the TC desorption degree increases. During the experiments, the lowest TC desorption was observed from BCV and BCH, whereas the highest was from non-modified BC. Vitamin C modified the surface of biochar by introducing additional aromatic rings and the protonation of basic groups, and thus TC was the most strongly adsorbed on this material. BCH is positively charged in the entire pH range and, as a result, TC is also strongly bounded with its particles via electrostatic attractive forces. Desorption measurements carried out with NaOH and HCl showed that HCl was definitely a more effective desorbing agent than NaOH. It allowed the desorption of more than 73% of TC from the surface of BC, which is a good preparation of the material for re-use.

## 4. Conclusions

This paper investigates the chemical modification of sunflower husk biochar by hydrogen peroxide, silver nanoparticles or vitamin C, and it defines the impact of these treatments on BC adsorption efficiency relative to tetracycline.

The properties of biochar, such as specific surface area, porosity, surface charge density, and the content of the functional surface group, changed after the modification. All modifiers reduced the specific surface area, micropore volume, and total pore volume of biochar. However, they increased the amount of acidic groups and basic groups on the BC surface. The performed treatments also influenced the surface charge density of adsorbents. Hydrogen peroxide made the BC surface charge more positive, whereas vitamin C made it more negative.

The kinetics of the TC adsorption process was well-described by the pseudo-second order equation, whereas equilibrium adsorption data was best fitted to the Redlich-Peterson model. Vitamin C improved the tetracycline adsorption to the greatest extent as well as lowering TC desorption. The TC adsorption efficiency for BCV was 47.75%. In contrast, the efficiency for non-modified biochar equaled 34.17%. The synthesized materials can be regenerated. An amount greater than 73% of TC was desorbed from the surface of BC using HCl.

The presented results showed that the obtained upgraded biochars have a good potential for the purification of aqueous media from tetracycline.

## Figures and Tables

**Table 1:** Adsorption modelling equations.

Equation	Formula	References
Pseudo-first order (PFO)	$\ln\left(q_{e}-q_{t}\right)=\ln q_{e}-k_{1}\cdot t$ (7)	[26]
Pseudo-second order (PSO)	$\frac{t}{q_{t}}=\frac{1}{k_{2}\cdot q_{e}^{2}}+\frac{t}{q_{e}}$ (8)	[27]
Particle internal diffusion model (IPD)	$q_{t}=k_{D}\cdot t^{\frac{1}{2}}+b$ (9)	[28]
Langmuir isotherm	$q_{e}=\frac{Q_{m}K_{L}C_{e}}{1+K_{L}C_{e}}$ (10)	[29]
Langmuir-Freundlich isotherm	${\left(\frac{C_{e}}{A_{m}}\right)}^{m}={\left(\frac{1}{A_{m}K_{LF}}\right)}^{m}+{\left(\frac{C_{e}}{A_{m}}\right)}^{m}$ (11)	[30]
Redlich-Peterson isotherm (R-P)	$ln(K_{RP}\frac{C_{e}}{q_{e}}-1)=\beta lnC_{e}+lna_{RP}$ (12)	[31]

q_t_ (mg/g)—the TC removal capacity at time t (min); q_e_ (mg/g)—the TC removal capacity at equilibrium; k_1_ (1/min) and k_2_ (g/mg·min)—the reaction rate constants; k_D_ (g/mg·min^1/2^)—the IPD rate constant; b (mg/g)—a layer thickness; C_e_ (mg/L)—the equilibrium concentration of TC in the solution; Q_m_ (mg/g)—the maximum number of TC in the monomolecular layer; K_L_ (L/mg)—the constant related to the affinity of the adsorbate for active sites; K_LF_, (L/mg)—related to the affinity of the adsorbate for active sites; A_m_ (mg/g)—the amount of the available surface sites; m—the parameter determining the shape of the energy distribution function; K_RP_ [L/mg]—the Redlich–Peterson adsorption capacity constant; a_RP_ [(L/mg)^β^]—the Redlich–Peterson isotherm constant; β—the exponent between 0 and 1.

**Table 2:** Properties of sunflower husks and biochar (M—the moisture content; HHV—the higher heating value; ED—the energy density).

Sample	M [%]	Ash [%]	H/C	O/C	(O+N)/C	HHV [MJ/kg]	ED [%]
Sunflower husks	9.38	2.43	0.12	0.84	0.85	16.31	2.01
Biochar	0.49	35.4	0.04	0.02	0.04	32.75	

**Table 3:** Surface chemistry and porosity of biochars (S_BET_—the specific surface area; V_m_—the micropore volume; V_t_—the total pore volume, D—the average pore diameter; TOC—the total organic carbon, BC—the non-modified biochar; BCV—the vitamin C-biochar; BCH—the H_2_O_2_-biochar; BCA—the Ag-NPs-biochar).

Sample	S_BET_ [m^2^/g]	V_m_ [cm^3^/g]	V_t_ [m^2^/g]	D [nm]	TOC [%]	Acidic Groups [mmol/g]	Basic Groups [mmol/g]
BC	7.02	0.004	7.98	3.54	82.15	2.90	3.20
BCV	1.30	0.001	3.40	36.37	82.92	3.10	3.40
BCH	2.19	0.002	4.07	5.11	80.72	5.20	6.90
BCA	0.10	0.001	3.84	17.68	82.64	4.00	5.20

**Table 4:** Functional groups of biochar determined by FTIR.

FTIR Adsorption Peak (cm^−1^)	Vibrations	References
3432	O-H stretching–carboxylic acid, hydroxyl groups and phenol	[21]
2922	C-H stretching–methyl and methylene groups	[40]
1611	C=O stretching–carboxylic and lactone groups and C=C stretching in aromatic rings	[30]
1384	N-O stretching–pyridinic-N oxide	[41]
1164	C-O stretching–saturated ester	[42]
1107	C-O-C stretching–fatty ether	[42]
1050	C-N and C=C stretching–secondary amines and aromatic structure	[41]
880	aromatic C–H stretching	[40]
811	aromatic C–H stretching	[40]
668	aromatic C–H stretching	[40]
580	chloride and CO_3_ = groups	[14]

**Table 5:** Kinetic and isotherms parameters for TC adsorption on modified and non-modified biochars.

Equation	Parameter	BC	BCV	BCH	BCA
PFO	q_e_ [mg/g]	5.10 ±0.49	7.28 ±0.34	6.67 ±1.16	6.96 ±0.29
	k_1_ [1/min]	0.11 × 10^−2^ ±0.02 × 10^−2^	0.28 × 10^−2^ ±0.17 × 10^−2^	0.12 × 10^−2^ ±0.09 × 10^−2^	0.21 × 10^−2^ ±0.16 × 10^−2^
	R^2^	0.69	0.61	0.86	0.85
PSO	q_e_ [mg/g]	6.99 ±0.88	7.85 ±0.61	7.14 ±0.35	7.55 ±0.54
	k_2_ [g/mg·min]	0.29 × 10^−2^ ±0.13 × 10^−2^	0.54 × 10^−2^ ±0.05 × 10^−2^	0.41 × 10^−2^ ±0.15 × 10^−2^	0.47 × 10^−2^ ±0.02 × 10^−2^
	R^2^	0.99	0.99	0.99	0.99
IPD	b [mg/g]	3.55 ±0.72	4.29 ±0.99	3.98 ±0.88	4.11 ±0.92
	k_D_ [g/mg·min^1/2^]	0.19 × 10^−2^ ±0.13 × 10^−2^	0.33 × 10^−2^ ±0.17 × 10^−2^	0.29 × 10^−2^ ±0.16 × 10^−2^	0.32 × 10^−2^ ±0.16 × 10^−2^
	R^2^	0.47	0.38	0.36	0.41
Langmuir	K_L_ [L/mg]	1.02 × 10^−2^ ±0.98 × 10^−2^	2.09 × 10^−2^ ±0.69 × 10^−2^	1.24·10^−2^ ±0.87 × 10^−2^	1.64 × 10^−2^ ±0.47 × 10^−2^
	Q_m_ [mg/g]	7.26 ±1.61	10.56 ±2.82	7.93 ±1.16	8.09 ±2.19
	R^2^	0.94	0.98	0.95	0.98
Langmuir-Freundlich	K_LF_ [L/mg]	3.36 × 10^−2^ ±0.21 × 10^−2^	3.88 × 10^−2^ ±0.14 × 10^−2^	3.64 × 10^−2^ ±0.19 × 10^−2^	3.42 × 10^−2^ ±0.96 × 10^−2^
	A_m_ [mg/g]	6.17 ±0.46	9.13 ±0.15	8.09 ±0.21	8.39 ±0.22
	m	1.17 ±0.17	1.19 ±0.11	1.17 ±0.14	1.21 ±0.05
	R^2^	0.94	0.98	0.95	0.98
Redlich-Peterson	K_R_ [L/mg]	2.81 ±0.29	8.65 ±0.08	4.17 ±0.21	6.33 ±0.22
	a_R_ [(L/mg)^β^]	2.32 ±0.55	5.66 ±0.95	4.81 ±0.24	3.74 ±0.51
	β	0.24 ±0.06	0.39 ±0.13	0.29 ±0.09	0.31 ±0.05
	R^2^	0.98	0.99	0.98	0.99

**Table 6:** The degrees of TC desorption.

Sample	Deionized Water			HCl	NaOH
	5	7	9		
BC	18.20%	19.24%	20.42%	66.28%	3.69%
BCV	11.71%	11.56%	14.76%	73.82%	8.23%
BCH	12.15%	12.75%	15.45%	71.33%	4.83%
BCA	13.82%	16.67%	17.22%	62.88%	2.95%

## Data Availability

Not applicable.
